# Detecting the presence of fish farm-derived organic matter at the seafloor using stable isotope analysis of phospholipid fatty acids

**DOI:** 10.1038/s41598-017-05252-w

**Published:** 2017-07-11

**Authors:** Daniel J. Mayor, Nia B. Gray, Giannina S. I. Hattich, Barry Thornton

**Affiliations:** 1 National Oceanography Centre, Southampton, SO14 3ZH United Kingdom; 2 James Hutton Institute, Aberdeen, AB15 8QH United Kingdom; 3 Oceanlab, University of Aberdeen, AB41 6AA Aberdeen, United Kingdom; 4 GEOMAR, Helmholtz Centre for Ocean Research Kiel, Wischhofstr. 1-3, 24148 Kiel, Germany

## Abstract

The expansion of global aquaculture activities is important for the wellbeing of future generations in terms of employment and food security. Rearing animals in open-exchange cages permits the release of organic wastes, some of which ultimately reaches the underlying sediments. The development of rapid, quantitative and objective monitoring techniques is therefore central to the environmentally sustainable growth of the aquaculture industry. Here, we demonstrate that fish farm-derived organic wastes can be readily detected at the seafloor by quantifying sediment phospholipid fatty acids (PLFAs) and their carbon stable isotope signatures. Observations across five farms reveal that farm size and/or distance away from it influence the spatial distribution of the generated organic wastes and their effect on benthic bacterial biomass. Comparison to the isotopic signatures of fish feed-derived PLFAs indicates that 16:0 and 18:1(n-9) are potential biomarkers for fish farm-derived organic wastes. Our results suggest that stable isotope analysis of sediment PLFAs has potential for monitoring the environmental performance of aquaculture activities, particularly given the increasing prevalence of terrigenous organic matter in aquaculture feed stocks because it is isotopically district to marine organic matter.

## Introduction

Global aquaculture production must double by 2050 if current per-capita consumption levels of aquatic, animal-derived protein are to be sustained as the human population reaches 9 billion^1, 2^. Achieving the necessary level of growth and intensification of aquaculture activities within environmentally sustainable limits represents one of this global industry’s major bottlenecks^2, 3^.

Marine finfish aquaculture, and in particular the production of Atlantic salmon, *Salmo salar*, has seen explosive growth over the past 3 decades and this species alone now has an estimated global value >US$14bn^4^. Rearing finfish in mesh cages allows the release of particulate organic wastes that can subsequently accumulate in the underlying and surrounding sediments. The extent to which the seafloor becomes organically enriched is determined by a variety of interacting environmental parameters, including distance away from the farm, current speed, water depth, and farm size^5–9^. Monitoring, understanding and mitigating the effects of aquaculture-derived organic wastes on the structure and biogeochemical functioning of the receiving communities are key to the expansion and intensification of this global industry^2^.

The effects of fish farming activities on the underlying sediments can be quantified by examining changes in the structure of benthic macrofaunal communities, which exhibit predictable and readily discernible changes in response to organic enrichment^10^. This sensitive and reproducible approach has been widely-adopted^6, 11^, but is nevertheless time consuming and thus expensive^12^. Alternative approaches to quantify the fate of farm-derived organic wastes include the analysis of sediment bulk stable isotope signatures^13–15^, fatty acid compositions^16–18^, or combinations thereof^19^. Most recently, high-throughput sequencing metabarcoding of environmental samples has been proposed as a rapid and reproducible, albeit not quantitative, method for discerning the impacts of fish farming activities on benthic ecosystems^20–22^.

Compound-specific stable isotope analysis (CSSIA) of sediment phospholipid fatty acids (PLFAs) potentially represents an additional, quantitative method for rapidly determining the benthic impacts of aquaculture. Biological membranes are largely comprised of phospholipids and the constituent PLFAs have been widely used to provide insight into the biomass and structure of microbial communities for several decades^23–26^. The advent of CSSIA techniques has enabled the carbon isotopic (δ^13^C) signatures of individual PLFAs to be accurately determined, providing insight into the source of organic matter used by microorganisms^27^. This is particularly useful in the context of marine finfish aquaculture, where the feedstock may be isotopically distinct from benthic microbial communities, e.g. because it is derived from terrestrial sources^28^ and/or from fishmeal/oil harvested in other regions of the globe^29^. Here, we present data on the concentrations, relative abundances and isotopic signatures of sediment PLFAs in the vicinity of five Scottish fish farms alongside information on the composition of feed pellets. Our study explores the overarching hypothesis that aquaculture-derived organic matter at the seafloor, and the influences of fish farm size (maximum consented biomass, MCB), average current speed and distance from the cage edge, can be detected using CSSIA of sediment PLFAs.

## Results

### Composition of feed pellets

Feed pellets contained 48.17 ± 1.03% carbon (% dry weight ± SD, n = 5) and 6.11 ± 0.07% nitrogen, with bulk isotopic values of −22.56 ± 0.26 and 9.75 ± 0.17 for δ^13^C and δ^15^N respectively. The major phospholipid fatty acids and their isotopic signatures are presented in Table 1.

**Table 1:** Composition and isotopic signatures of phospholipid fatty acids (PLFAs) (average ±SD) in salmon feed pellets (Skretting Label Rouge, 11 mm).

PLFA	Mol %	δ^13^C (per mil)
14:0	0.92 ± 0.15	−18.93 ± 1.88
16:1(n-7)	1.51 ± 0.25	−21.37 ± 1.68
16:0	29.74 ± 0.49	−27.91 ± 0.09
18:2(n-6, 9)	17.04 ± 0.21	−29.49 ± 0.50
18:1(n-9)	23.37 ± 0.63	−31.33 ± 0.36
18:0	4.57 ± 0.09	−23.53 ± 0.96
20:5(n-3)	4.13 ± 0.23	−23.74 ± 1.78
22:6(n-3)	18.71 ± 0.66	−26.44 ± 0.16

### Bacterial biomass

**Figure 1:**
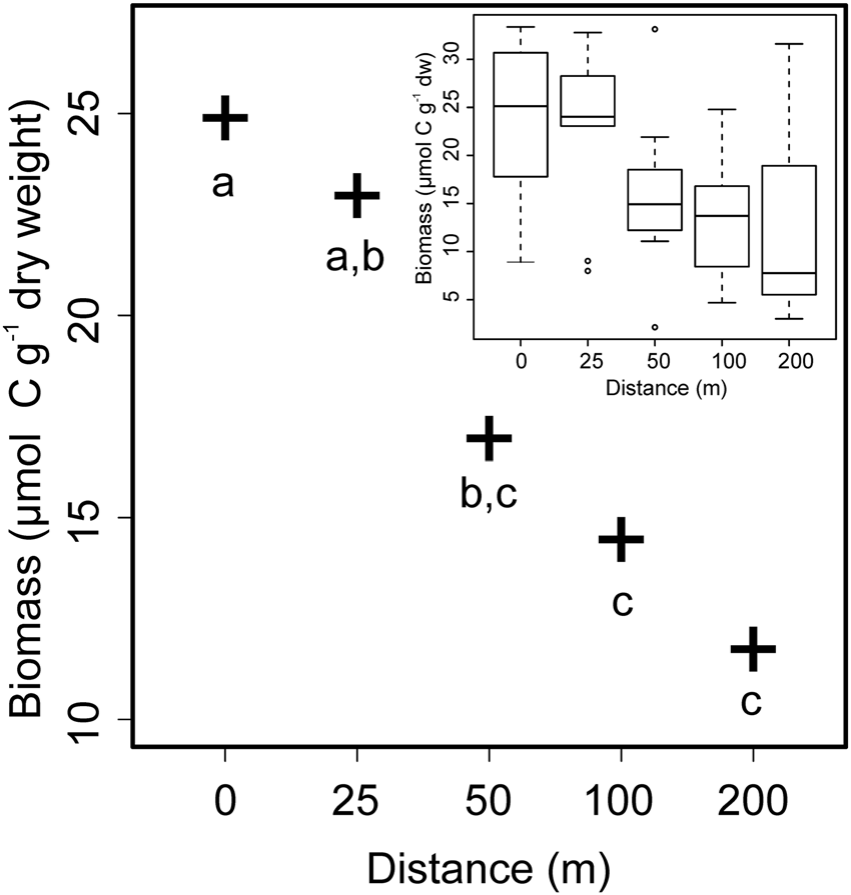
Model-predicted estimates of bacterial biomass in the surficial sediments at 5 Scottish fish farms illustrating the effects of distance (m) from the cage edge. Common letters (**a**,**b**,**c**) denote that distances are not significantly different from each other (p > 0.05). Inset boxplot presents the analyzed data.

PLFA-derived estimates of bacterial biomass, which were analysed using a linear mixed-effects (LME) model that included Farm ID as a random effect (L. Ratio = 13.45, df = 1, p < 0.001), declined with increasing distance from the cage edge (Fig. 1; L. Ratio = 21.26, df = 4, p < 0.001). The effects of average current speed (L. Ratio = 0.02, df = 1, p = 0.873) and MCB (L. Ratio = 1.39, df = 1, p = 0.237) did not improve the model’s performance and were therefore removed. Model coefficients are presented in the Supplementary Information Table S1.

### Surficial sediment PLFA profiles and their isotopic signatures

The absolute concentrations (nmol [g dry sediment]^−1^), percentage abundance (mol %) and isotopic composition (δ^13^C) of individual PLFAs in relation to MCB and distance are presented in Supplementary Figures S1–S6.

**Figure 2:**
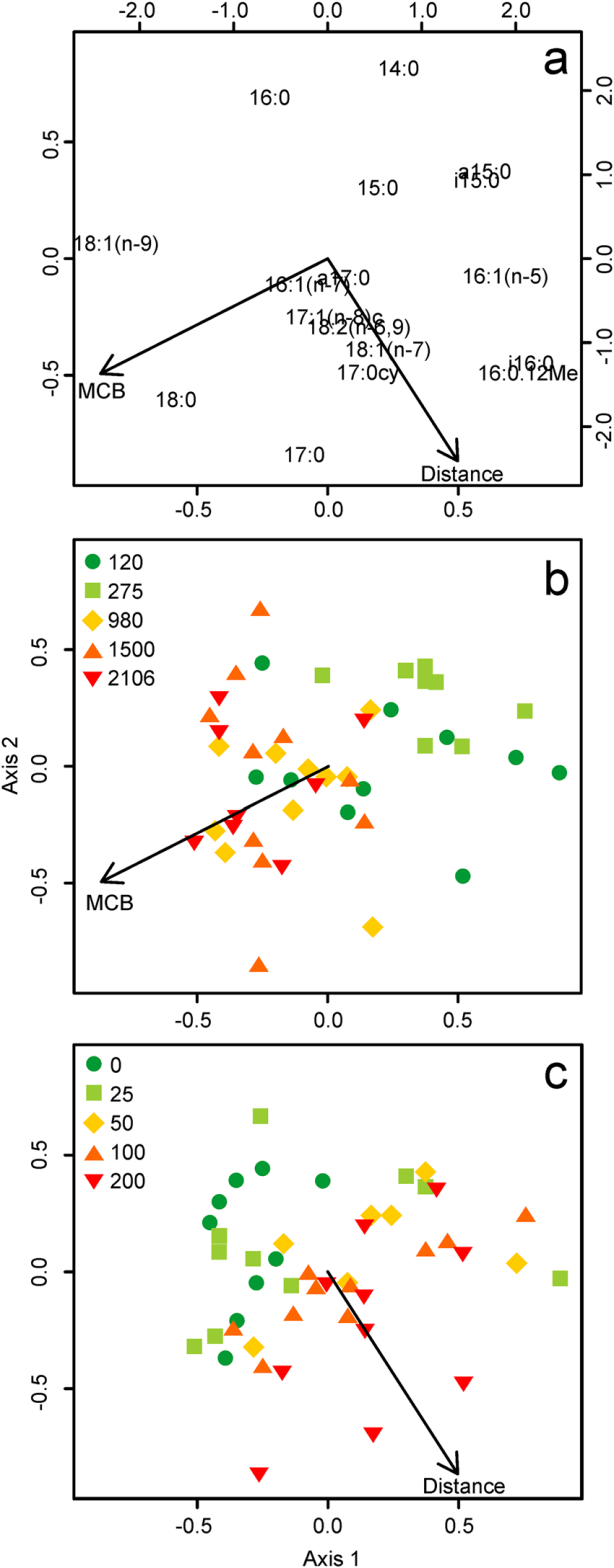
Redundancy analysis distance triplot of phospholipid fatty acid (PLFA) profiles (mol %) extracted from surficial sediments around 5 fish farms in Scotland. The upper panel (**a**) visualizes how the explanatory variables MCB (maximum consented biomass, tonnes) and distance from the cage edge (m), plotted on the primary axes, were related to the 17 PLFAs (‘species’), plotted on the secondary axes. The lower panels illustrate how the composition of PLFAs in each individual sediment sample (‘sites’, n = 46) was related to MCB (**b**) and Distance (**c**).

The redundancy analysis (RDA) model of the mol % PLFA data included MCB (F = 4.81, df = 43, p < 0.001) and distance (F = 3.00, df = 41, p = 0.003), explaining 16% of the variance in the data; 8% was solely attributable to MCB and 4% to distance. An interaction between MCB and distance was not apparent (F = 0.70, df = 39, p = 0.687). The first axis of the RDA triplot (Fig. 2a) had strong positive loadings of PLFAs (‘species’) with 15 and 16 carbon atoms, including the bacterial biomarkers a15:0, i15:0 and i16:0. Axis 1 had negative loadings of 18:0 and 18:1(n-9). The second axis of the mol % RDA triplot had positive loadings of 14:0 and 16:0 and negative loadings of 17:0. The effect of MCB (Fig. 2a) was spread across the negative ends of axes 1 and 2; samples (‘sites’) collected from larger farms are located towards the bottom left hand corner of the plot and vice versa (Fig. 2b). Overall, larger farms were positively correlated with the relative abundances of the PLFAs 18:0 and 18:1(n-9) and negatively correlated with a15:0 and i15:0. The effect of increasing distance (Fig. 2a and c) was positively correlated with axis 1 and negatively correlated with axis 2; samples collected at the cage edge appear towards the top left of the plot and move towards the bottom right as distance increased (Fig. 2c). Sediments from the cage edge were strongly associated with the PLFA 16:0 whereas those collected 200 m away from the fish farm were correlated with the PLFAs 17:0cy, 18:1(n-7) and to a lesser extent, 17:0, i16:0 and 16:0.12Me.

**Figure 3:**
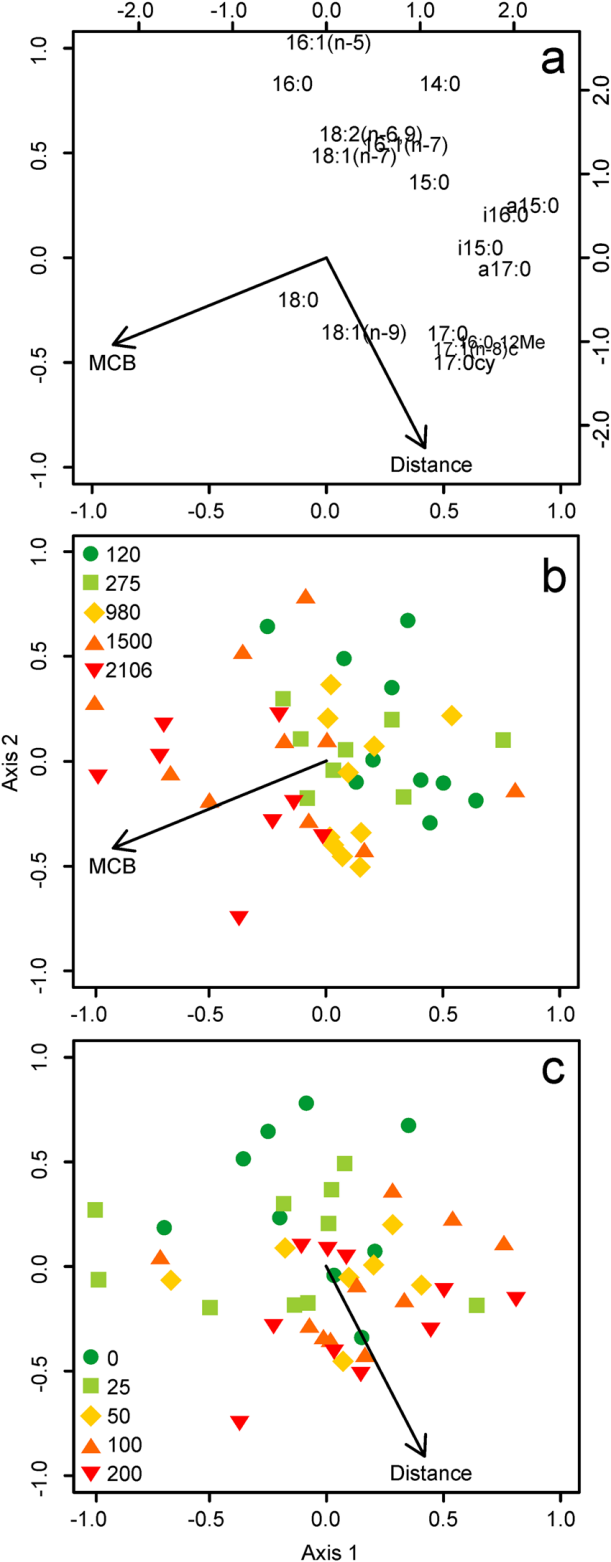
Redundancy analysis distance triplot of phospholipid fatty acid (PLFA) profiles (δ^13^C) extracted from surficial sediments around 5 fish farms in Scotland. The upper panel (**a**) visualizes how the explanatory variables MCB (maximum consented biomass, tonnes) and distance from the cage edge (m), plotted on the primary axes, were related to the 17 PLFAs (‘species’), plotted on the secondary axes. The lower panels illustrate how the isotopic signatures of PLFAs in each individual sediment samples (‘sites’, n = 46) were related to MCB (**b**) and Distance (**c**).

The RDA model of the δ^13^C data was similar to that of the mol % data; it included MCB (F = 5.01, df = 43, p < 0.001) and distance (F = 3.23, df = 41, p = 0.004), but not their interaction (F = 0.96, df = 39, p = 0.445). The explanatory variables collectively explained 16% of the variance in the mol% data (‘species’), with 8% and 4% being exclusively attributable to MCB and distance respectively. Axis 1 of the δ^13^C RDA triplot (Fig. 3a) was dominated by strong positive loadings of odd-chain length and branched PLFAs, including a15:0, i16:0, a17:0, 16:0.12Me, i15:0, 17:1(n-8), 17:0cy and 17:0. The second axis was positively loaded by 16:1(n-5), 16:0 and 14:0, and negatively loaded by 17:0cy, 17:1(n-8), 16:0.12Me, 17:0 and 18:1(n-9). MCB was negatively correlated with the majority of the PLFA δ^13^C values (Fig. 3a and b), and in particular a15:0, i16:0, i15:0 and a17:0, indicating that there was a general tendency for δ^13^C values of these moieties to become increasingly negative, i.e. depleted in ^13^C, as MCB increased. Distance was positively correlated with 17:0cy, 17:1(n-8), 16:0.12Me, 17:0 and 18:1(n-9) (Fig. 3a and c), indicating that their δ^13^C values increased with distance away from the cage edge. The PLFAs 16:1(n-5), 16:0 and 14:0 showed strong, negative correlations with distance; these moieties became more isotopically depleted as distance from the farm increased.

## Discussion

This study quantified the concentrations of PLFAs and their isotopic signatures in the sediments underlying five fish farms in Scotland. Our data demonstrate that PLFA-derived estimates of bacterial biomass, along with the composition and δ^13^C signatures of sediment PLFAs all changed significantly with increasing distance from the cage edge and/or the size of a farm.

All of the locations sampled have been used to farm Atlantic salmon, *Salmo salar*, for ≥5 years, but the 3 largest sites (MCB ≥980 tonnes) were holding Atlantic cod, *Gadus morhua*, at the time of sampling. It is thus possible that the observed effect of farm size could, in part, be attributed to the difference in farmed species. However, the observed changes in the composition and isotopic signatures of sediment PLFAs in response to MCB (Figs 2b and 3b, respectively) occurred progressively from small to large farms, rather than abruptly at the boundary between the salmon and cod farms as may be expected if these two were in some way distinct. If the presence of cod had exerted a major influence on the relative abundance of sediment PLFAs we would have also expected to see this emerge when the data were re-plotted to show the effect of distance but no such effect was apparent (Figs 2c and 3c respectively). Rather, the effect of distance on the relative abundance of PLFAs and their δ^13^C values can also be seen as a gradual shift in both cases (Figs 2c and 3c respectively). We therefore reason that culture-species identity effects were not responsible for the major trends observed in our data.

We refrain from attempting to attribute the observed changes in the PLFA data to the relative abundances of specific groups of microorganisms because of the difficulties associated with the non-specificity of individual PLFAs^30^. Equally, we avoid over-interpretation of the PLFA δ^13^C values because of the numerous processes that influence these^31, 32^. Instead, we focus our discussion around the general patterns that emerge in the data and, more broadly, the potential utility of CCSIA of sediment PLFAs as a tool for robustly detecting changes at the seafloor and associating these with fish farm-derived organic wastes.

### Fish farm size effects

Previous studies investigating how fish farm size affected the biology and chemistry of the underlying sediments reported that its effect, where present, depended upon the response variable of interest. In many cases, no effects were discernible and in others, its effects were either additive or dependent upon water column current speed^8, 9^. Our estimates of benthic bacterial biomass, derived from sediment concentrations of the biomarker PLFAs i15:0, ai15:0 and i16:0, showed no relationship with farm size (p = 0.237). The consistent levels of bacterial biomass across the range of farm sizes examined suggests that the consenting and management practices applied across these farms were sufficient to keep the deposition of organic wastes below the assimilative capacity of the biological communities in the receiving sediments. This finding agrees with the understanding that the sediment concentrations of organic matter at the sampled locations, determined by thermogravimetric analysis (‘loss on ignition’ at 525 °C), and total nitrogen concentrations are not affected by farm size^9^. It is further corroborated by a recent study which indicated that although fish farming affected the detailed bacterial community structure, it did not influence the overall abundance of the most abundant operational taxonomic units^21^.

By contrast, in our multivariate analyses we found that farm size was the principle variable explaining the relative composition (p < 0.001; Fig. 2b) and isotopic signatures of sediment PLFAs (p < 0.001; Fig. 3b). The association between farm size and the relative composition of the sediment PLFA pool was, in part, driven by the strong and positive correlations between the feed-derived PLFAs, 18:0 and 18:1(n-9) (Table 1), and farm size (Fig. 2a). Interestingly, the other major feed-derived PLFAs showed no clear association with farm size, and the relative abundances of many non-feed moieties were strongly negatively correlated (Fig. 2a). The isotopic signatures of sediment PLFAs were also principally influenced by farm size, but unlike the majority of PLFAs, the δ^13^C values of 18:0 and 18:1(n-9) remained largely unchanged across the different farm sizes, as indicated by the proximity of these PLFAs to zero on the primary x-axis of the RDA plot (Fig. 3a). The isotopic signature of 18:0 varied by ~10‰ between the feed pellets (δ^13^C = −23.53; Table 1) and the sediments (δ^13^C = −33.31; Supplementary Figures S3 and S6), suggesting considerable fractionation of this moiety, or perhaps more likely, that the 18:0 observed in sediments was derived from the elongation of shorter-chain, more depleted feed pellet-derived PLFAs such as 16:0 (δ^13^C = −27.91; Table 1), de novo biosynthesis and/or from other sources altogether^33–36^. By contrast, the strong similarity between the δ^13^C value of 18:1(n-9) in the feed-pellets (δ^13^C = −31.33; Table 1) and sediments (δ^13^C = −31.28; Supplementary Figures S3 and S6) suggests that the presence of this fatty acid in the sediment PLFA pool is directly related to the deposition of farm-derived organic wastes. We therefore propose three lines of evidence to indicate that the abundance of 18:1(n-9) and its isotopic signature are useful proxies for discerning the environmental fate of feed-derived wastes in the sediments beneath salmon farms: This compound is: (a) highly abundant in fish oil- and fishmeal-containing feed pellets (Table 1), (b) strongly associated with farm size (Fig. 2a), and (c) directly incorporated into the benthic PLFA pool. Furthermore, we suggest that 18:1(n-9) will serve as a useful biomarker for understanding the future effects of salmon farming, and perhaps other culture species, as 18:1(n-9) remains a major component of aquaculture feeds even when the marine oils are replaced with terrestrial oils^37, 38^.

The observed effects of farm size on both the relative composition and δ^13^C values of non-feed-derived PLFAs in the sediments could indicate that the structure and/or metabolic functioning of the benthic microbial communities differed across the investigated sites^27, 36, 39^. However, interpretation of these data in such a manner requires caution because the presence of feed-derived PLFAs influences the relative abundances of all other PLFAs. For example, the strong negative correlations between farm size and the proportional abundances of the generic bacterial biomarker PLFAs, a15:0 and i15:0, suggests that bacterial abundance in the vicinity of the farms sampled decreased as farm size increased. At first glance this appears to oppose our previous assertion that PLFA-derived estimates of bacterial biomass were not influenced by farm size (p = 0.237). These apparently contradictory results arise because the relative abundances of a15:0 and i15:0 are influenced by the strong correlations between farm size and 18:0 and 18:1(n-9), i.e. as the relative abundances of 18:0 and 18:1(n-9) increase with farm size, the relative abundances of a15:0 and i15:0 decrease, whereas their absolute concentrations remain unchanged in relation to farm size. Interpreting the strong effect of farm size on the relative abundance and isotopic signatures of non-feed-derived PLFAs also requires caution because of potential location-specific differences in characteristics of the seafloor that are correlated with farm size, rather than because of any direct effect of farm size-related differences in management practices. For example, farm size is potentially correlated with sediment grain size, a major determinant of bacterial community structure^40, 41^, because larger farms are only permitted in areas with elevated current speeds^42^.

### Distance from cage edge effects

The observed reduction in PLFA-derived estimates of bacterial biomass with increasing distance from the cage edge (p < 0.001; Fig. 1) agrees well with previous studies that have examined the response of benthic bacterial abundance to organic enrichment arising from aquaculture activities using direct counts^43–47^, fatty acid biomarkers^17^ and molecular techniques^43–48^. By contrast, the relative abundances of other putative bacterial biomarker PLFAs, e.g. i16:0, i16:0.12Me, 17:0cy, and 18:1(n-7), all increased with increasing distance (Figs 2a and S5). Similar effects of distance on the concentrations and relative abundances of bacterial fatty acids were previously reported in the sediments around a seabream farm in the Gulf of Eilat, Israel^17^. These apparently counterintuitive results are, again, most likely explained by disproportionally high concentrations of feed-derived PLFAs in close proximity to the farms (e.g. 16:0, 16:1(n-7), 18:0; Supplementary Figure S4), effectively lowering the relative abundance of other, bacteria-specific PLFAs in the sediments close to the cages^17^. This interpretation is supported by examination of the feed-derived PLFAs, 14:0, 16:0, 18:0 and 18:1(n-9) (Table 1), all of which were negatively correlated with distance (Fig. 2a), i.e. their concentrations and relative abundances were greatest at the cage edge sampling locations (Supplementary Figures S4 and S5). The major PLFA in the feed pellets was 16:0 and the strong negative correlation between the relative abundance of this moiety and distance suggests that it also a likely candidate biomarker for tracing the fate of farm-derived organic waste at the seafloor.

The isotopic signatures of many of the identified PLFAs were also correlated with distance (Figs 3a and S6). Some moieties became isotopically enriched (contained more ^13^C) with increasing distance (e.g. 17:0, 17:0cy, 18:1(n-9)), whereas others became depleted (e.g. 14:0, 16:0, 16:1(n-5)). Interpretation of these data is complex, not least because the isotopic signatures of individual PLFAs in the feed pellets varied by >12‰ (Table 1) and were generally isotopically depleted relative to the bulk organic carbon signature of the pellets (δ^13^C = −22.56‰). Nevertheless, the observation that the relative abundances and isotopic signatures of both 16:0 and 18:1(n-9) respectively decreased and increased with increasing distance from the sampled farms further supports our assertion that these compounds offer promise as useful biomarkers of fish farm-derived organic wastes.

The isotopic signatures of individual PLFAs in the feed pellets and sediments, and hence potential levels of isotopic fractionation, also showed a wide range of variance. The δ^13^C values of the PLFAs 14:0, 16:1(n-7) and 18:0 in the feed pellets were all ≥9‰ enriched relative to their respective values in the sediments (Table 1 and Supplementary Figures S3 and S6), suggesting that these moieties are subject to significant fractionation between their source (feed pellet) and sink (sediment). This could occur as a result of digestive and metabolic processes within the farmed fish^49^, and/or because of microbial metabolism^50, 51^. By contrast, the isotopic signature of 16:0 in the sediments (δ^13^C = −32.45; Supplementary Figures S3 and S6) was closer to the value observed in the feed (δ^13^C = −27.91; Table 1), suggesting that this moiety was subject to less fractionation prior to and during its incorporation into the sediments. It is not possible to mechanistically explain the observed patterns in the δ^13^C signatures presented herein. To do so would require information on the spatio-temporal variation in (1) the isotopic signatures of all the source substrates being delivered to the seafloor, e.g. the size and biochemical composition of feed pellets and inputs of marine and terrigenous organic matter are all likely to vary over the growth cycle, (2) the active metabolic pathways in the sediments, and (3) the relative importance of substrate dilution, degradation and fractionation. Nevertheless, the significant effect of distance in all of our presented analyses is consistent with various other studies that have examined spatial patterns of fish farm-derived organic wastes^5–9^. These observations collectively demonstrate that fish farming activities do effect changes at the seafloor that can be detected by CSSIA of sediment PLFAs. More importantly, however, the data presented herein reveal that concentrations of benthic bacterial biomass at 50 m from the cage edge were not statistically greater than those at further distances away from the farms investigated (p ≥ 0.087; Supplementary Table S1). This suggest that, at least in the context of Scottish sea-cage fish farming, existing management and farming practices typically confine the benthic footprint of effects to ≤50 m from the edge of a farm^8, 9^.

### Fish farm monitoring using PLFA techniques

The presented data illustrate that CSSIA is a potentially useful technique for detecting aquaculture-derived wastes at the seafloor. However, this method is not without limitations. It lacks the species-specific resolution provided by faunal monitoring techniques, and further work is required to validate the link between sediment PLFAs and the structure and biogeochemical functioning of the benthic communities. These shortfalls could be at least partially resolved by combining CSSIA of sediment PLFAs with high-throughput sequencing of environmental DNA, which has recently been demonstrated to faithfully reflect faunal-based indices routinely used in benthic monitoring activities^22^. We suggest that CSSIA of sediment PLFAs offers advantages over traditional benthic monitoring practices, which typically include the analysis of benthic macrofaunal community composition^6, 11^: The CSSIA of PLFAs is quantitative and highly reproducible^39^ and can be largely automated, generating data within only a few days of sample acquisition, relative to the weeks or months required to process the equivalent number of faunal samples^12^. Faunal analysis requires a high level of specific expertise. In contrast, the identification and quantification of individual PLFAs can be achieved with expertise that is more common across many analytical laboratories. The inclusion of information on the isotopic composition of the PLFAs provides further potential to quantitatively link the observed patterns at the seafloor to the inputs of farm-derived organic wastes^13–15^. This aspect of the method is particularly useful given the clear compositional and isotopic differences between the fish meal- and fish oil-rich feed stocks given to Atlantic salmon (Table 1) and the underlying sediments (Supplementary Figures S1–S6). We expect these differences to also occur as the proportion of terrestrial plant-derived material in aquaculture feed stocks increases^52^ because terrigenous organic matter is isotopically distinct to that produced in the oceans^28^. Indeed, the inclusion of terrigenous organic matter in feed stocks inadvertently provides a natural isotopic tracer for discerning the fate of the constituent carbon in marine ecosystems. We suggest that the identification and stable isotope analysis of specific terrigenous biomarker molecules offers further potential for readily and quantitatively tracing the fate of organic wastes arising from aquaculture activities in marine environments. The potential to quantify waste accumulation has benefits from an environmental perspective and may also help operators to further understand and refine their feeding practices within days of field sampling.

The highly significant effects of farm size and distance on the relative abundances and isotopic signatures of sediment PLFAs demonstrate that the inputs of fish farm-derived organic matter can be readily discerned using CCSIA techniques. The myriad processes that influence the composition and δ^13^C signatures of PLFAs remain poorly understood and currently confound our ability to provide a definitive and mechanistic interpretation of the observed changes. Nevertheless, the reported patterns did not arise by chance. Rather, we contend that they occurred, at least in part, because the relative input of fish-farm derived organic wastes as a carbon source to the benthos changed across the different farms and along the transects sampled. We therefore suggest that the analysis of sediment PLFAs offers potential for monitoring the effects of aquaculture on the surrounding environment. This cost-effective method is rapid, quantitative and objective. The available data indicate that 16:0 and 18:1(n-9) represent strong candidate biomarker molecules for understanding patterns of fish farm-derived organic enrichment at the seafloor. The inclusion of CSSIA further allows the fate of organic wastes in the surrounding environment to be determined, particularly in situations where feed stocks containing a significant proportion of terrigenous organic matter are used in the marine environment. We propose that CSSIA of sediment PLFAs in combination with other emerging techniques, e.g. high-throughput sequencing of environmental DNA, represents a useful addition, or even alternative, to more traditional faunal-based approaches.

## Methods

Single surficial (0–2 cm) sediment samples were collected with a 0.1 m^2^ day grab along two transects at 0, 25, 50, 100 and 200 m distance from the cage edge at 5 active fish farms within a region of Scotland in 2006. Exact sampling locations are omitted, as agreed with the farm operators prior to sampling. All samples were oven-dried (55 °C) and stored frozen (−18 °C) prior to analysis. The size of these farms, defined as the Maximum Consented Biomasses (MCB) of Atlantic salmon permitted on site at any given time, were 120, 275, 980, 1500 and 2106 tonnes. Hydrographic data for each site, collected by independent consultants, were provided by the farm operators. All of the sampled farms have a long (≥5 yrs) history of farming Atlantic salmon, and the 3 largest farms (≥980 tonnes) were holding Atlantic cod, *Gadus morhua*, at the time of the surveys (with cod MCB’s being 657, 990 and 1411 tonnes, respectively). Previous work conducted on samples from these locations concluded that observed patterns of sediment chemistry were not attributable to the species being cultured^9^. Representative salmon on-growing feed pellets (Skretting Label Rouge, 11 mm) were obtained from Skretting.

Concentrations and isotopic signatures of organic carbon and nitrogen in the salmon feed pellets were determined on pre-weighed aliquots of freeze-dried and homogenised material using a Flash EA 1112 Series Elemental Analyser connected via a Conflo III to a Delta^Plus^ XP IRMS (Thermo Finnigan, Bremen, Germany). The PFLAs within the sampled sediments and feed pellets were extracted and derivitized to produce fatty acid methyl esters (FAMEs) using well established methods^23, 53^. The concentrations and carbon isotope ratios of individual FAMEs were determined using a GC Trace Ultra with a combustion column attached via a GC Combustion III to a Delta V Advantage IRMS (all Thermo Electron, Bremen, Germany). Individual PLFAs were quantified by combining the area of their mass peaks, m/z = 44, 45 & 46, after background subtraction, and through comparison with a known internal standard (19:0) added to each sample^54^. Carbon isotope ratios of individual PLFAs were calculated with respect to Vienna-PDB (δ^13^Cv-_PDB_) through the use of a reference gas injected before every sample and traceable to International Atomic Energy Agency reference material NBS 19 TS-Limestone. Long term measurement of the Indiana University reference material hexadecanoic acid methyl ester #1 (certified δ^13^C_VPDB_ value = −30.74 ± 0.01‰) gave a value of −30.80 ± 0.45‰ (mean ± sd, n = 81).

Bacterial biomass was estimated from the concentration of the bacterial biomarker PLFAs: i15:0, ai15:0 and i16:0^25, 55, 56^ assuming that these PLFAs make up 10% of total bacterial PLFAs and that there is 0.056 g C PLFA/g C biomass^57^. Estimated bacterial biomass data were analysed using linear mixed-effects (LME) regression techniques that incorporated farm identity as a random effect to account for the inherent correlations between data collected within each farm^9^. The fixed structure of the initial model included farm size, average current speed and distance from the cage edge as explanatory variables and all possible 2-way interaction terms. Non-significant terms were removed via hierarchical backwards selection using the likelihood ratio (L. Ratio)^8, 58^. Preliminary data exploration revealed 4 potential outliers (≥3 standard deviations from the mean) and the data were therefore Box-Cox transformed (λ = 0.1121) prior to analysis. Re-analysis of the untransformed data with the 4 outliers removed yielded the same model structure as the analysis of Box-Cox transformed data. The untransformed data analysis is presented to facilitate data interpretation. The influence of farm size, distance from the cage edge and average current speed on the relative abundance and isotopic signatures of sediment PLFAs was examined using redundancy analysis (RDA). A permuted (n = 9999) forwards model selection procedure was used to determine the significance of individual model terms^31, 59^. All statistical analyses were conducted in the ‘R 3.2.0’ programming environment^60^ using the ‘nlme’^61^ and ‘vegan’^62^ packages.

## Electronic supplementary material

Supplementary Information
