# Supplementary material for: Detecting the presence of fish farm-derived organic matter at the seafloor using stable isotope analysis of phospholipid fatty acids
Source: Sci Rep. 2017 Jul 11;7:5146. doi: 10.1038/s41598-017-05252-w (PMC5506006; doi:10.1038/s41598-017-05252-w)
Supplement: Supplementary file 1 — Supplementary Information [file 41598_2017_5252_MOESM1_ESM.pdf]

Detecting the presence of fish farm-derived organic matter at the seafloor using stable isotope analysis of phospholipid fatty acids

Daniel J. Mayor [1,2,3,*], Nia B. Gray [2], Giannina S. I. Hattich [4], Barry Thornton [2]

[1] National Oceanography Centre, Southampton, SO14 3ZH, United Kingdom

[2] James Hutton Institute, Aberdeen, AB15 8QH, United Kingdom

[3] Oceanlab, University of Aberdeen, AB41 6AA, United Kingdom

[4] GEOMAR, Helmholtz Centre for Ocean Research Kiel, Wischhofstr. 1-3, 24148 Kiel, Germany

* Corresponding author. E-mail: dan.mayor@noc.ac.uk

Supporting information

Table S1. Model output for Linear Mixed-Effects analysis of bacterial biomass.

Fig. S1. Effect of farm size on concentrations of individual sediment phospholipid fatty acids (PLFAs).

Fig. S2. Effect of farm size on the relative abundances of individual sediment PLFAs.

Fig. S3. Effect of farm size on the carbon isotopic signatures of individual sediment PLFAs.

Fig. S4. Effect of distance on concentrations of individual sediment PLFAs.

Fig. S5. Effect of distance on the relative abundances of individual sediment PLFAs.

Fig. S6. Effect of distance on the carbon isotopic signatures of individual sediment PLFAs.

Supplementary Table S1. Model output for Linear Mixed-Effects analysis of bacterial biomass. Random effect (a), intra-class correlation (b) and fixed-effects (c), showing coefficients ± SE and t-values with p-value in parentheses.

a)		σ				
	Farm ID	6.054593				
	Residual	6.219214				
b)	Intra-class correlation	0.48659				
c)		Distance 0 m	Distance 25	Distance 50	Distance 100	Distance 200
	(Intercept)	24.66036 ± 3.758683<br>6.560905<br>(<0.001)	-	-	-	-
	d25	-1.9255 ± 3.305643<br>-0.58249<br>(0.5641)	-	-	-	-
	d50	-7.94265 ± 3.440417<br>-2.30863<br>(0.0272)	-6.017144 ± 3.052032<br>-1.971521<br>(0.0568)	-	-	-
	d100	-10.4357 ± 3.265569<br>-3.19568<br>(0.003)	-8.510214 ± 2.865840<br>-2.969535<br>(0.0054)	-2.493069 ± 2.963226<br>-0.841336<br>(0.4060)	-	-
	d200	-13.1531 ± 3.265569<br>-4.02782<br>(0.0003)	-11.227611 ± 2.865840<br>-3.917738<br>(0.0004)	-5.210467 ± 2.963226<br>-1.758376<br>(0.0877)	-2.717398 ± 2.781317<br>-0.977018<br>(0.3355)	-

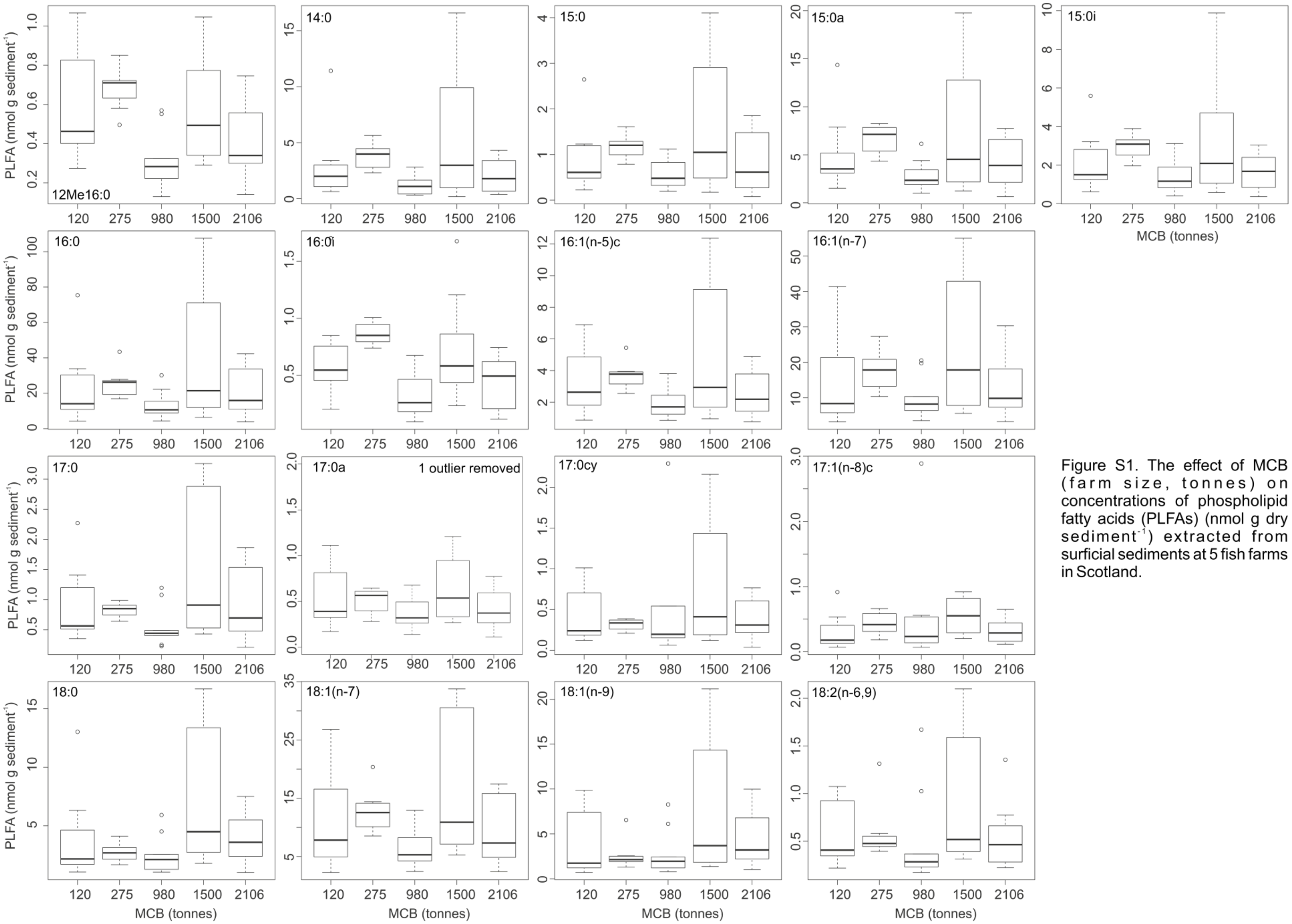

Figure S1. The effect of MCB (farm size, tonnes) on concentrations of phospholipid fatty acids (PLFAs) (nmol g dry sediment^{-1}) extracted from surficial sediments at 5 fish farms in Scotland.

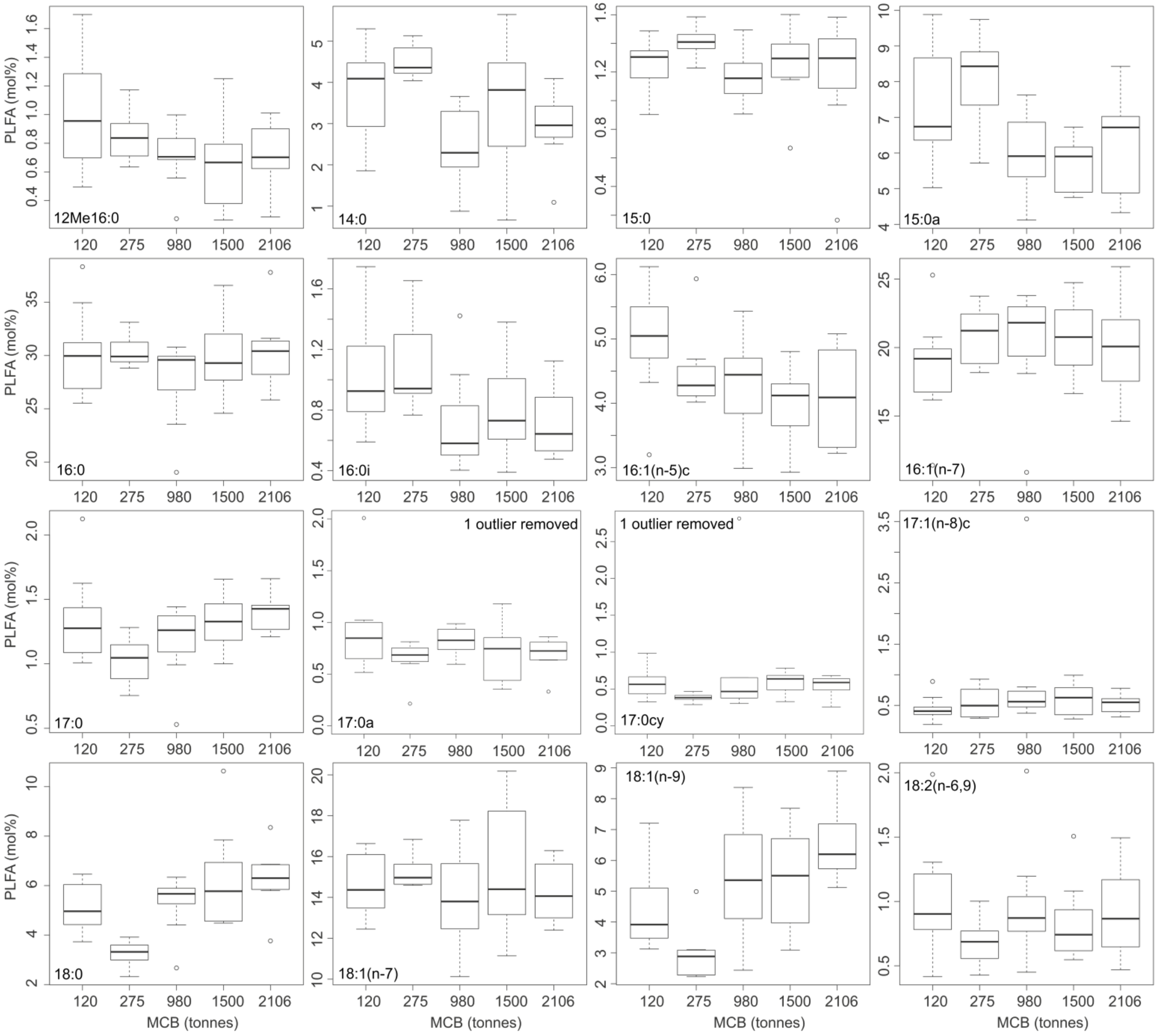

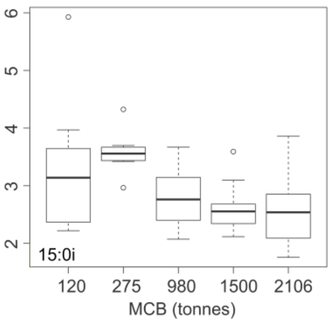

Figure S2. The effect of MCB (farm size, tonnes) on the relative abundance of phospholipid fatty acids (PLFAs) (mol %) extracted from surficial sediments at 5 fish farms in Scotland.

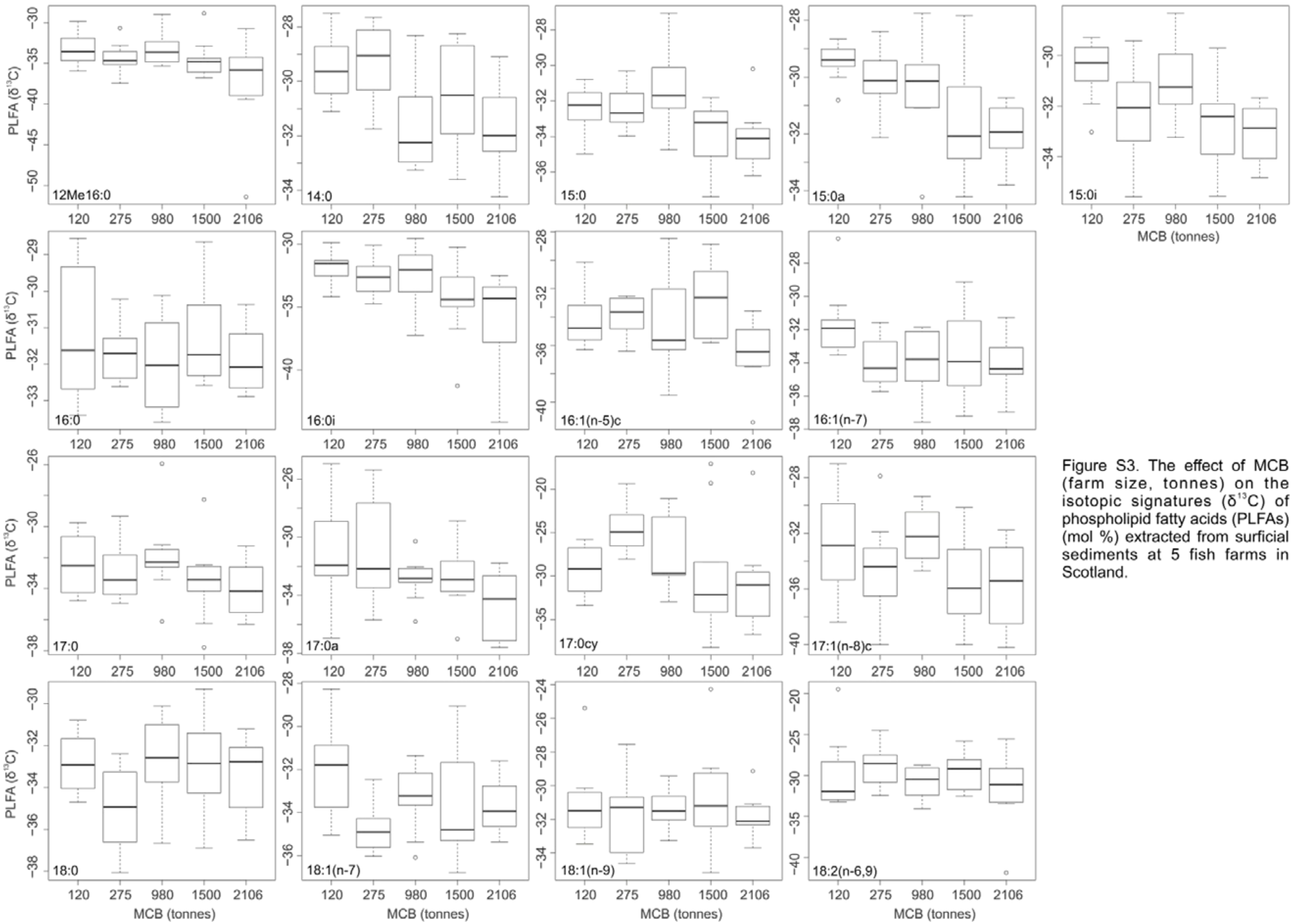

Figure S3. The effect of MCB (farm size, tonnes) on the isotopic signatures ($\delta^{13}C$) of phospholipid fatty acids (PLFAs) (mol %) extracted from surficial sediments at 5 fish farms in Scotland.

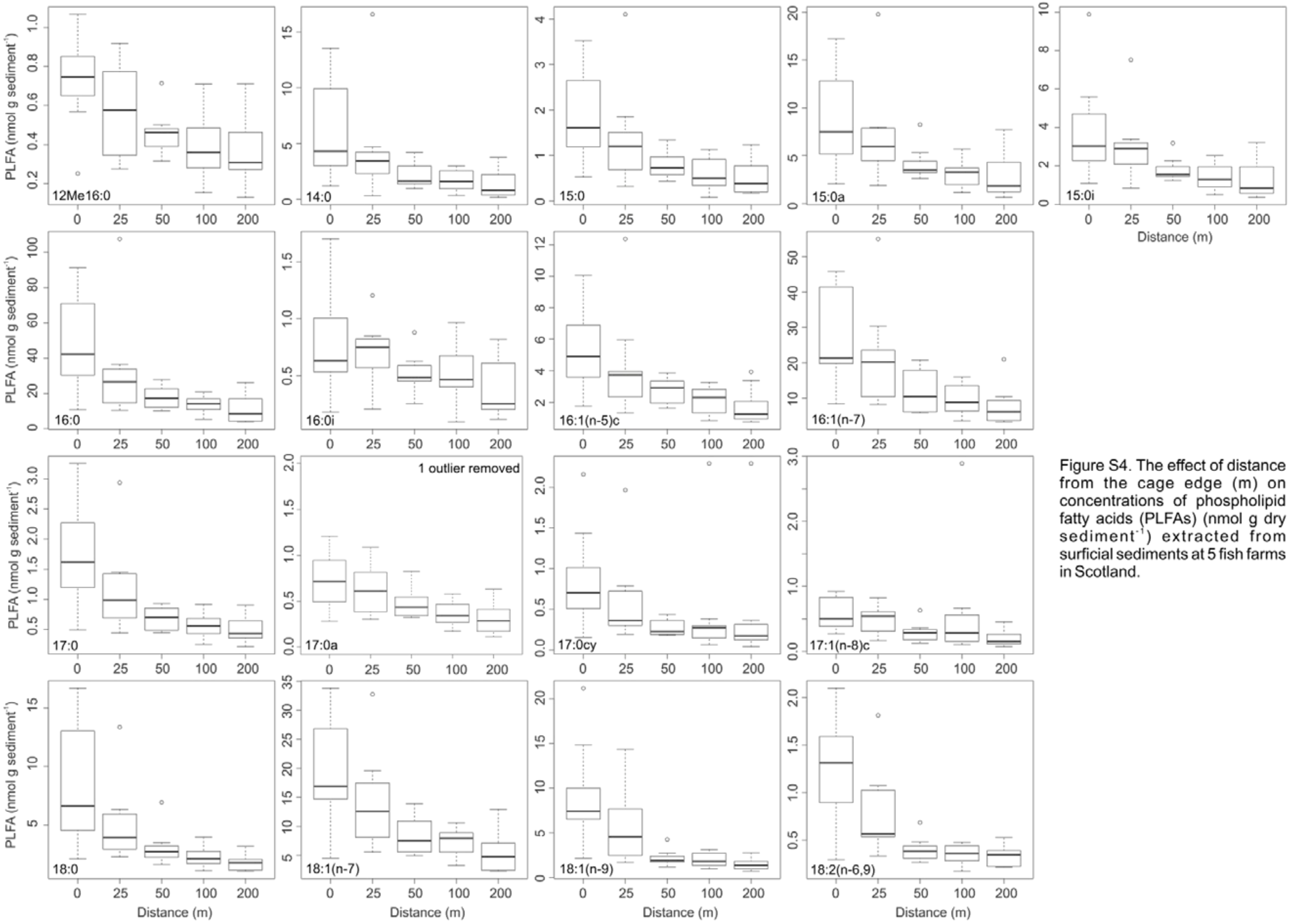

Figure S4. The effect of distance from the cage edge (m) on concentrations of phospholipid fatty acids (PLFAs) (nmol g dry sediment^{-1}) extracted from surficial sediments at 5 fish farms in Scotland.

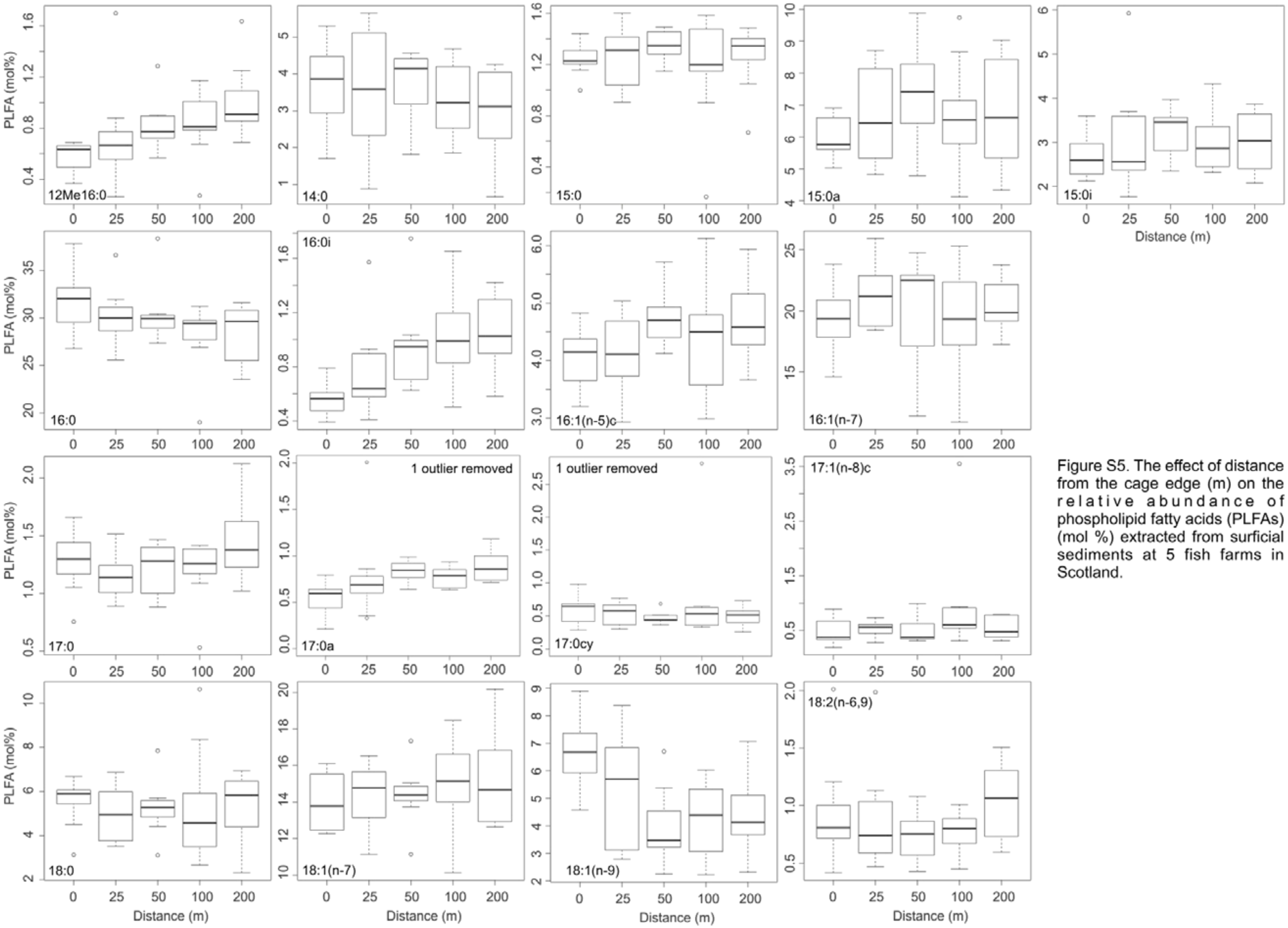

Figure S5. The effect of distance from the cage edge (m) on the relative abundance of phospholipid fatty acids (PLFAs) (mol %) extracted from surficial sediments at 5 fish farms in Scotland.

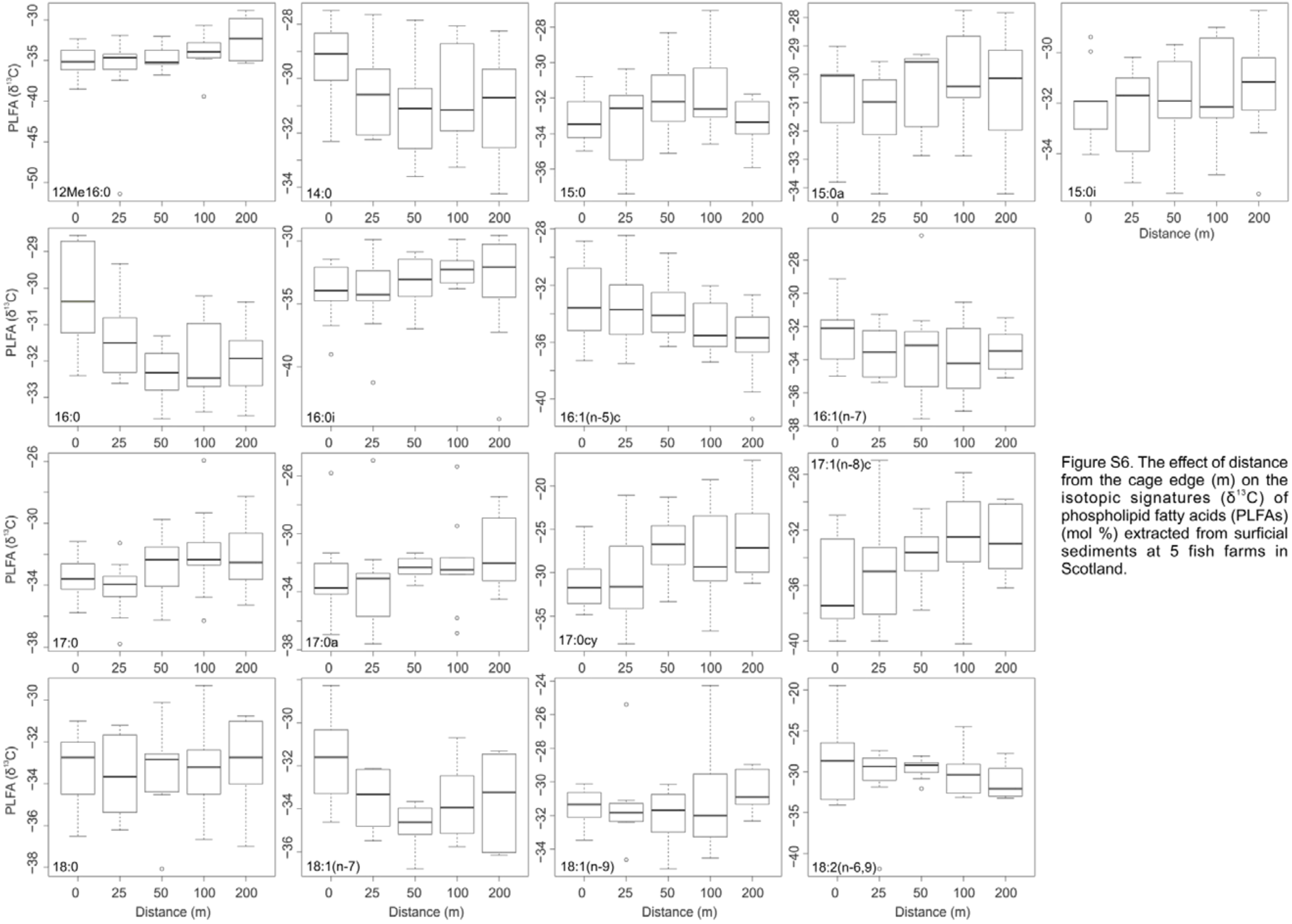

Figure S6. The effect of distance from the cage edge (m) on the isotopic signatures ($\delta^{13}C$) of phospholipid fatty acids (PLFAs) (mol %) extracted from surficial sediments at 5 fish farms in Scotland.
